# Determinants of maternal health care and birth outcome in the Dande Health and Demographic Surveillance System area, Angola

**DOI:** 10.1371/journal.pone.0221280

**Published:** 2019-08-22

**Authors:** Edite Vila Nova Rosário, Manuel Carmo Gomes, Miguel Brito, Diogo Costa

**Affiliations:** 1 Centro de Investigação em Saúde de Angola (CISA), Bengo, Angola; 2 Instituto de Saúde Pública da Universidade do Porto (ISPUP), Porto, Portugal; 3 Faculdade de Ciências, Universidade de Lisboa, Lisboa, Portugal; 4 Health and Technology Research Center (H&TRC), Escola Superior de Tecnologia da Saúde de Lisboa, Instituto Politécnico de Lisboa, Lisboa, Portugal; African Population and Health Research Center, KENYA

## Abstract

**Objectives:**

Maternal health care improvement and reduction of maternal and child mortality are priorities of the global health agenda. In Angola, maternal mortality remains high and the risk of pregnancy-related death was 1 in 32 during 2015. This study aims to identify demographic and social factors influencing antenatal care and health facility delivery among women in Dande and to understand their impact on birth outcomes.

**Methods:**

This study is based on community–based longitudinal data collected by the Dande Health and Demographic Surveillance System between 2009 and 2015. Data on pregnancy outcomes (10,289 outcomes of 8,066 women) were collected for all reported pregnancies, including sociodemographic information, health services utilisation and women’s reproductive history. Logistic regression was used to investigate the determinants of birth outcomes, antenatal care attendance and institutionalised delivery.

**Findings:**

Of the 10,289 pregnancy outcomes, 98.5% resulted in live births, 96.8% attended antenatal care, and 82.5% had four or more visits. Yet, 50.7% of the women delivered outside a health facility. Antenatal care attendance was a determinant of birth outcomes (stillbirth: unadjusted OR = 0.34 95% CI = 0.16–0.70; abortion: OR = 0.07 95% CI = 0.04–0.12). Older women, with lower education, living at a greater distance of a health facility and in rural areas, were less likely to use maternal health care. Having had previous pregnancies, namely resulting in live births, also decreased the likelihood of health care utilization by pregnant women.

**Conclusions:**

The study identifies relevant social determinants for the utilisation of antenatal care, place of delivery and their impact on birth outcome, thereby providing insight on how best to address inequities in health care utilization.

## Introduction

Maternal and child mortality reduction and improvements in women and children health care are priorities of the global health and international development agendas. They were integrated into the Millennium Development Goals (MDG) set for accomplishment until 2015, and remain in the Sustainable Development Goals (SDG) agenda for 2030 [1, 2].

By the end of the MDG era, several indicators showed that progress achieved across regions and countries regarding reduction in maternal mortality was below the established target and was geographically unequal [3]. The MDG5 target, aiming maternal health improvement, included a 75% decline in global maternal mortality ratio (MMR) between 1990 and 2015, but the observed reduction was only of 44% [3]. Regional disparities are evident, as deaths occurring in developing countries accounted for 99% of all maternal deaths, with the sub-Sahara African region alone bearing 66% of the burden [1, 3]. In 2015, the global lifetime risk of maternal mortality was approximately 1 in 180, but in sub-Saharan Africa it was estimated to be 1 in 36, in sharp contrast with approximately 1 in 4,900 in developed countries [3].

Most maternal deaths are preventable as they stem from insufficient health support during pregnancy and delivery [3–5]. Improvements in maternal and infant health require, although not exclusively, the provision of accessible reproductive health care and skilled attendance at delivery [4, 6, 7]. In 2013, the global coverage of skilled birth attendants (SBA) was 74% and the percentage of women with the recommended four or more antenatal care (ANC) visits was 64% [8], against 52% and 49% in sub-Saharan Africa [9]. The reduction of inequalities is a keystone in the new ‘leaving no one behind’ strategic framework for action in the SDG era [3, 10].

Angola had a reduction of 58.9% in MMR between 1990 and 2015. However, by the end of the MDG period, maternal mortality remained high, with a MMR estimated at 477 deaths per 100,000 live births and a risk of pregnancy-related death of 1 in 32 [1].

In Angola, the Multiple Indicator Cluster Survey (MICS) report of 2015–16 provides some information on the use of reproductive health care [11], but the overall data on maternal health are scarce and there is a paucity of research on the subject. The percentage of women attending at least one ANC visit, was 82% in 2015, very close to the global coverage of 83% in 2013 [8]. Nevertheless, more than half of the women still gave birth at home, with only 46% of births taking place in a health facility [11], far less than 71% of births globally attended by skilled personnel in 2014 [12].

This abandoning or lack of access to the health system at the time of delivery is poorly understood as there is a general absence of studies on the determinants of maternal health care utilisation in Angola and their impact on birth outcomes. Although previous studies report the determinants of maternal care utilization and their importance in other Sub-Saharan African countries [3, 13–15], this research is needed in Angola, since evidence can be context dependent. Such evidence is required to understand and reduce health inequities [16].

This study aims to identify demographic and social factors influencing the utilisation of ANC and health facilities for delivery among women in the Dande Health and Demographic Surveillance System (HDSS) area between 2009 and 2015, as well as their impact on birth outcomes.

## Materials and methods

### Setting

We have analysed data from the HDSS located in Dande Municipality, Bengo Province, located about 60 km to the north-east of Luanda. The area, with approximately 4,700 km^2^, covers all the 70 hamlets located in three of the five municipality communes—Caxito, Mabubas and Ucua—plus a smaller portion of hamlets in the Barra do Dande and Kicabo communes [17]. Urban areas cover approximately 77% of the total population (27 hamlets). We adopted the classification of the Angolan National Institute of Statistics (INEA), which defines urban areas as including all province capitals (as Caxito), and towns with 2.000 or more inhabitants, having basic infrastructures like schools, health centres, and paved main roads. Rural areas are mainly dispersed settlements [18] whose socioeconomic conditions, accessibilities and population lifestyle differ markedly from urban living. In general, the accesses are rudimentary for both the rural and urban areas covered: there are only two main paved roads crossing the HDSS area and unpaved dirt roads constitute the main routes, constraining access to some communities, especially in the rainy season [17].

Until 2015, the public health facilities that served the HDSS area were a general hospital, a municipal hospital, a maternal and infant health centre, two health centres and 10 primary health centres. A private health centre, run by the catholic church, was also settled in the surveillance area. Maternal health (pre and postnatal care) was available in eight of those facilities, including the private health centre. Birth delivery support was available in three facilities: two hospitals, both located in the city of Caxito, and the maternal and infant health centre located at the urban area periphery. In most of these health facilities, material and human resources are scarce, causing frequent constraints to both routine and emergency care services that are offered. Additional information on the HDSS area, including health human resources has been published before [17].

The HDSS was implemented in 2009 and an initial census, performed between August 2009 and March 2010, established the baseline study population, registering 15,579 households with 59,635 residents. Women comprised 51% of the population (30,414). The household’s geographical coordinates were collected using a geographical positioning system (GPS). After the initial census, update rounds (UR), consisting of periodic house-to-house visits, collected data on households’ characteristics and demographic events, such as pregnancies, births, deaths and migrations.

Between 2010 and 2014, women aged 15–49 years living in the Dande HDSS represented on average 23.1% of the total population and the total fertility rate (number of children born or likely to be born to a woman in her lifetime if she were subject to the prevailing rate of age-specific fertility in the population) for the same period was on average 4.3 [17].

### Sampling and data collection

Data collection was carried out during nine update rounds, performed between August 2009 and December 2015, using structured questionnaires administered at each household (S1 and S2 Text). Data on pregnancy outcomes were collected for all reported pregnancies, including information on the women, their ANC attendance, birth outcome (live births, stillbirths or abortions) and place of delivery (health centre, hospital or outside a health facility). Information was collected for 10,289 pregnancy outcomes from 8,066 women. In the ninth round (February to October 2015), several questions were added to the inquiry form, namely the place where the women conducted ANC visits (public or private), the number and timing of visits, if the women had been pregnant before, and if so, how often, and the resulting number of live births out of those pregnancies. This added information was collected for 2,187 pregnancy outcomes.

The data were collected by 18 local fieldworkers who had received training on explaining the HDSS objectives, techniques for conducting interviews and instructions on how to complete the questionnaires. Six supervisors assured the quality of work: four checked all the completed forms and monitored the fieldwork, whereas two identified and corrected errors and incongruences at the data centre. Six data clerks inserted all data following a double entry system. A manual was provided with instructions regarding all procedures.

Women’s basic sociodemographic data were retrieved from the HDSS databases, namely birth date, literacy (can read and write), years of schooling and place of residence.

### Variables

Dependent variables–Three dependent variables were selected for the analyses: The first was ‘**birth outcome’**, with three categories: live birth (98.5% of 10,289 birth outcomes), stillbirth (foetal death with a gestation period of 28 or more weeks, 0.9% of birth outcomes), and abortion (foetal death with less than 28 weeks of gestation, comprising 0.6% of birth outcomes). In statistical analysis where independent variables had relatively small samples (n≤ 2,187), stillbirths and abortions were lumped into a single category of adverse outcomes. The second dependent variable was ´**ANC attendance**’ (yes or no), being yes if women attended at least one appointment. The third was ‘**place of delivery**’–in a health facility *versus* delivery outside a health facility.

Independent variables–These were women’s age (continuous variable), education (school attendance, number of years of schooling and literacy), their geographic location (approximate road distance of their households to health facilities, commune of residence and rural or urban location), number of ANC visits, gestational age at first ANC visit, previous pregnancies and respective outcomes, and number of live children.

### Data analysis

Data were entered and analysed for both descriptive and inferential statistics using the Statistical Package for Social Sciences (SPSS) software, version 23.0. Analysis of variance and post-hoc tests were used as appropriate to compare means of continuous variables. The effects of predictive variables over dependent variables were studied by binary logistic regression, except when the dependent variable was pregnancy outcome and independent variables had large sample sizes (>10,000) allowing for the splitting of pregnancy outcome into three categories, which were studied by multinomial logistic regression. Associations were studied by bivariate and multivariate analysis, with the computation of, respectively, crude and adjusted odds ratios (OR). In multivariate analysis, we used an exploratory model building approach, as independent variables were selected for being suspected predictors and were entered in block. We have first studied the association with independent variables with n>10,000 observations, doing a separate analysis for variables with smaller samples, namely those related to women´s past pregnancy experience. Post-hoc power analysis was done with G*Power 3 [19] when there was a suspicion of a Type II error.

### Ethical considerations

This study was approved by the Ethics Review Committees of the Ministry of Health of Angola and the Institute of Public Health of the University of Porto. As an HDSS implies frequent visits to households to update information, verbal consent was deemed appropriate for monitoring demographic events. Participation was voluntary. All forms used in the HDSS were approved and registered in the Angolan National Institute of Statistics, namely the birth outcome form.

## Results

A total of 10,289 birth outcome forms were collected between 2009 and 2015. The mean age of women who gave birth was 26.5 years old (± 7.21), with 3,596 (34.9%) of them at ages deemed of higher risk pregnancy (< 20 and > 35 years old) [20]. Women´s education was generally low: the mean number of years spent at school was 2.57 (± 3.48), but more than half of the participants had no education (55.5%). From a total of 10,289 pregnancies, 84.3% of women were living in an urban area and 73.4% at a distance to the nearest facility of less than 2 km. The majority of women attended an ANC visit during pregnancy (96.8%). Information regarding the number of visits and gestational age at first ANC visit was available for, respectively, 1,747 and for 2,007 women. The mean number of ANC visits was 5.84 (± 2.27) and 82.5% reported to have had four or more ANC visits. The first ANC visit occurred mainly in the first trimester of pregnancy (63.7%). Nearly one-sixth of women (15.7%) was *nulligravida* and 84.3% had been pregnant before. For *gravida*, the mean number of pregnancies was 3.21 (±2.99) and the mean number of previous live births was 3.01 (±2.87). About 16.7% of women with previous pregnancies had never had a live birth. Despite high adherence to ANC visits, when asked about the place of delivery only 49.3% reported having had an institutionalized birth (Table 1).

**Table 1:** Descriptive statistics of the sample.

Variable	Categories	n (%)
**Women´s age** (years)	<20 years old	1,975 (19.2)
(N = 10,289)	20–34 years old	6,697 (65.1)
	35–44 years old	1,543 (15.0)
	>45 years old	74 (0.7)
Mean ± sd; min-max	26.5 ± 7.21 (11–57)	
**Women´s education**	No education	5,776 (55.5)
**(Completed years of schooling)**	≤ 4 years of schooling	1,647 (16.4)
**(**N = 10,039)	5–8 years of schooling	2,004 (20.0)
	9–12 years of schooling	727 (7.2)
	>12 years of schooling	85 (0.8)
Mean ± sd; min-max	2.57 ± 3.48 (0–13)	
**Women´s Literacy**	Yes	2,990 (63.9)
(N = 4,680)	No	1,690 (36.1)
**Place of residence**	Urban	8,676 (84.3)
(N = 10,289)	Rural	1,613 (15.7)
**Approximate distance to**	<2 km	7,550 (73.4)
**a health facility** (N = 10,289)	2–5 km	1,656 (16.1)
	6–10 km	293 (2.8)
	11–15 km	227 (2.2)
	>15 km	563 (5.5)
**ANC attendance**	Yes	9,759 (96.8)
(N = 10,084)	No	325 (3.2)
**Nr. of ANC visits** (N = 1,747)	<4	305 (17.5)
	4–8	1,112 (63.6)
	≥9	330 (18.9)
Mean ± sd; min-max	5.84 ± 2.27 (1–10)	
**Type of ANC provider**	Public	1,895 (91.0)
(N = 2,083)	Private	188 (9.0)
**Gestational age at 1**^**st**^ **ANC**	1^st^ trimester	1278 (63.7)
**visit** (N = 2,007)	2^nd^ trimester	688 (34.3)
	3^rd^ trimester	41 (2.0)
**Have been pregnant**	Yes	1,843 (84.3%)
**before** (N = 2,187)	No	344 (15.7%)
**Nr. previous pregnancies**	0 times	344 (15.8)
(N = 2,180)	1 time	251 (11.5)
	2 to 3 times	661 (30.3)
	4 to 5 times	557 (25.6)
	6 or more times	367 (16.8)
Mean ± sd; min-max	3.21 ± 2.99 (0–13)	
**Parity (Nr. of previous live**	0 live birth	364 (16.7)
**Births)** (N = 2,178)	1 live birth	297 (13.6)
	2 to 3 live births	670 (30.8)
	4 to 5 live births	549 (25.2)
	6 or more live births	298 (13.7)
Mean ± sd; min-max	3.01 ± 2.87 (0–11)	
**Place of delivery**	Health facility delivery	4,962 (49.3)
(N = 10,059)	Home delivery	5,097 (50.7)
**Birth outcomes** (N = 10,289)	Live birth	10,131 (98.5)
	Stillbirth	97 (0.9)
	Abortion	61 (0.6)

sd = standard deviation; min = minimum; max = maximum; ANC = Antenatal care

### Demographic, social, and obstetric determinants associated with birth outcome

The odds of a pregnancy ending in stillbirth was associated with both ANC attendance and with the place of residence: having at least one ANC visit decreases the odds of stillbirth (unadjusted OR = 0.34, 95% Confidence Interval, CI = 0.16–0.70) and women living in rural areas have higher odds of delivering a stillbirth (OR = 2.11, 95% CI = 1.35–3.29) than those in urban settings. These associations were not significant though when the OR was adjusted for factors like place of delivery, distance to a health facility, women´s age and education (Table 2). Proximity to a health facility tended to decrease the odds of a stillbirth (unadjusted OR’s for <2 km, 2–5 km, 6–10 km, and 11–15 km were, respectively, 0.43, 0.54, 1.35, and 1.50), although the protective effect of proximity was significant only for living at < 2 km in the bivariate analysis (Table 2). The odds of a stillbirth decreased for women with more years of school education (unadjusted OR = 0.96, *p =* 0.22) and tended to increase with women’s age (OR = 1.02, *p =* 0.19), but none of these associations was significant. In a post-hoc power analysis (one-sided test, with α = 0.05), the probability of not incurring in a Type II error, when comparing the risk of stillbirth between women who were one standard deviation apart in years of age was only 35.2%, while in years of schooling was 53.3%. To achieve a power of at least 80%, a sample size four times greater would be required for ages and twice greater for years of schooling.

**Table 2:** Adjusted and unadjusted Odds Ratios (OR) of stillbirth (top table) and abortion (bottom) against predictor variables, from multinomial logistic regression. Values of OR>1 indicate increased risk of stillbirth or abortion.

	**n stillbirths /** **n pregnancies (%)**	**Unadjusted OR (95% CI)**	***p***	**Adjusted OR (95% CI)**	***p***
ANC attendance					
No	8/325 (3.4%)	1		1	
Yes	87/9759 (0.9%)	0.34 (0.16–0.70)	<0.01	0.57 (0.25–1.29)	0.18
Place of residence					
Urban	70/8676 (0.8%)	1		1	
Rural	27/1613 (1.7%)	2.11 (1.35–3.29)	<0.01	1.59 (0.84–3.03)	0.16
Place of delivery					
Health facility	47/4962 (0.9%)	1		1	
Outside health facility	48/5097 (0.9%)	1.00 (0.67–1.9)	0.99	1.27 (0.83–1.95)	0.27
Km to health facility					
<2 Km	58/7550 (0.8%)	0.43 (0.22–0.84)	0.01	0.64 (0.27–1.53)	0.32
2–5 Km	16/1656 (1.0%)	0.54 (0.24–1.19)	0.13	0.80 (0.30–2.15)	0.66
6–10 Km	7/293 (2.4%)	1.35 (0.51–3.58)	0.55	1.93 (0.67–5.55)	0.22
11–15 Km	6/227 (2.6%)	1.50 (0.54–4.19)	0.44	1.44 (0.52–4.02)	0.49
>15 Km	10/563 (1.8%)	1		1	
Education (nr. years of schooling)	96/10239 (0.9%)	0.96 (0.90–1.02)	0.22	0.99 (0.93–1.06)	0.80
Women's age (years)	97/10289 (0.9%)	1.02 (0.99–1.05)	0.19	1.02 (0.99–1.04)	0.31
	**n abortions /** **n pregnancies (%)**	**Unadjusted OR (95% CI)**	***p***	**Adjusted OR (95% CI)**	***p***
ANC attendance					
No	19/325 (3.4%)	1		1	
Yes	41/9759 (0.4%)	0.07 (0.04–0.12)	<0.01	0.07 (0.04–0.13)	<0.01
Place of residence					
Urban	43/8676 (0.5%)			1	
Rural	18/1613 (1.1%)	2.29 (1.32–3.97)	<0.01	1.92 (0.98–3.79)	0.06
Place of delivery					
Health facility	20/4962 (0.4%)	1			
Outside health facility	41/5097 (0.8%)	2.00 (1.17–3.42)	0.01	1.30 (0.74–2.31)	0.36
Km to health facility					
<2 Km	47/7550 (0.6%)	0.87 (0.31–2.41)	0.78	3.57 (1.11–11.45)	0.03
2–5 Km	7/1656 (0.4%)	0.59 (0.17–2.02)	0.40	2.46 (0.63–9.56)	0.20
6–10 Km	1/293 (0.3%)	0.48 (0.05–4.33)	0.51	0.93 (0.10–8.73)	0.95
11–15 Km	2/227 (0.9%)	1.25 (0.23–6.89)	0.80	1.11 (0.20–6.23)	0.91
>15 Km	4/563 (0.7%)	1		1	
Education (nr. years of schooling)	61/10239 (0.6%)	0.91 (0.83–0.99)	0.03	0.94 (0.85–1.04)	0.22
Women's age (years)	61/10289 (0.6%)	1.00 (0.97–1.04)	0.99	0.99 (0.95–1.02)	0.41

ANC = Antenatal care¸ OR (95% CI) = Odds Ratio (95% Confidence Intervals)

The odds of abortion were found to decrease when there was ANC attendance (OR = 0.07, 95% CI = 0.04–0.12), both in bi and multivariate analysis. Living in rural areas increases the odds of abortion (unadjusted OR = 2.29, 95% CI: 1.32–3.97), moreover abortion was also associated with the place of delivery (unadjusted OR = 2.00, 95% CI: 1.17–3.42) (Table 2). The odds of abortion tend to decrease for women with more school education (unadjusted OR = 0.91, 95% CI: 0.83–0.99) and no association was found between odds of abortion and women’s age (OR = 1.00, *p =* 0.99).

All variables in Table 2 had sample sizes n>10,000. The association with women’s obstetric history was examined separately, using variables with smaller sample sizes, namely, the existence of at least one past pregnancy (yes/no; n = 2,187) and, if yes, having already experienced an adverse pregnancy outcome (yes/no; n = 1,836), and number of children alive (data for n = 2,178 women) (Table 3). Because age is a likely confounder in pregnancy history, crude and age-adjusted OR’s were estimated for these variables. Women who had past pregnancies were more likely to have an adverse outcome, but the association lacks statistical significance (unadjusted OR = 2.44, *p =* 0.39), and a similar result is observed after adjusting for age (OR = 1.99, *p =* 0.54). Women who had experienced a past adverse outcome had twice the odds of having a new one, but again the association was not significant (crude OR = 2.07, *p =* 0.23) (Table 3). Apparently sample sizes were not large enough to reject the null hypothesis in these associations, given the small numbers of adverse outcomes (14 in 2187 women)–a post-hoc power analysis estimates that the power to associate an adverse outcome with women who had past pregnancies was 26%, and with those who had past adverse outcomes was only 7% (two-sided tests, α = 0.05). The number of live children was positively correlated with the occurrence of an adverse pregnancy outcome, but this association was also not significant (OR = 1.21, *p =* 0.08).

**Table 3:** Unadjusted and age adjusted Odds Ratios (OR, 95% CI, p) for having an adverse pregnancy outcome, not attend ANC, and deliver outside a health facility, against three explanatory variables related with women’s history of pregnancy.

	Women (n)		To have an adverse outcome (OR 95% CI)	*p*	Not attend ANC (OR 95% CI)	*p*	Outside health facility (OR 95% CI)	*p*
Previously pregnant								
No	344		1		1		1	
Yes	1843	Unadj	2.44 (0.32–18.69)	0.39	2.44 (1.06–5.56)	0.04	2.94 (2.27–3.85)	<0.001
		Adjust	1.99 (0.23–17.65)	0.54	1.37 (0.56–3.45)	0.49	3.13 (2.38–4.17)	<0.001
Past adverse outcomes								
No	1510		1		1		1	
Yes	326	Unadj	2.07 (0.63–6.77)	0.23	1.56 (0.91–2.63)	0.11	1.03 (0.81–1.32)	0.78
		Adjust	2.01 (0.61–6.59)	0.25	1.43 (0.84–2.44)	0.19	1.04 (0.81–1.32)	0.76
Nr. of live children	2178	Unadj	1.21 (0.98–1.51)	0.08	1.27 (1.16–5.00)	<0.001	1.18 (1.14–1.23)	<0.001
		Adjust	1.28 (0.94–1.75)	0.12	1.22 (1.08–1.39)	0.003	1.30 (1.22–1.39)	<0.001

ANC = Antenatal care; Unadj = Unadjusted; Adjust = Adjusted; OR (95% CI) = Odds Ratio (95% Confidence Intervals)

### Demographic and social determinants associated with the utilisation of ANC services

Several factors significantly increase the risk of not having an ANC attendance. Namely, living at a greater distance to a health facility, living in a rural area, and having lower education, regardless of whether the association is seen from a bivariate or a multivariate viewpoint (Table 4). The association is particularly strong for rural areas (OR = 6.03, 95% CI = 4.81–7.55) and those living more than 6 km off a health facility (OR = 9.36 95% CI = 6.33–13.85, for 11–15 km away). The odds of not attending an ANC visit is also higher for older women, increasing 1.02 (95% CI = 1.00–1.03) per year of age. Having passed more years in school, on the contrary, decreases 0.83 the odds of non-attendance per additional year at school (95% CI = 0.79–0.88). Absence of literacy also increases the risk of no ANC attendance, although this variable becomes non-significant when the OR is adjusted for number of years of schooling.

**Table 4:** Adjusted and unadjusted Odds Ratios (OR) of ANC attendance against predictor variables, from binomial logistic regression. Values of OR>1 indicate increased risk of no ANC.

**Variables**	**n attending ANC /** **n pregnancies (%)**	**Unadjusted OR (95% CI)**	***p***	**Adjusted OR (95% CI)**	***p***
**Place of residence**					
Urban	8348/8509 (98.1%)	1		1	
Rural	1411/1575 (89.6%)	6.03 (4.81–7.55)	<0.001	2.95 (2.15–4.05)	<0.001
**Distance to health facility**					
< 2 km	7263/7414 (98.0%)	1		1	
2–5 km	1574/1612 (97.6%)	1.16 (0.81–1.66)	0.42	1.19 (0.83–1.72)	0.35
6–10 km	262/288 (91.0%)	4.77 (3.09–7.37)	<0.001	3.24 (2.06–5.10)	<0.001
11–15 km	185/221 (83.7%)	9.36 (6.33–13.85)	<0.001	3.17 (1.99–5.03)	<0.001
>15 km	475/549 (86.5%)	7.49 (5.59–10.05)	<0.001	2.61 (1.79–3.81)	<0.001
**Literacy**					
Yes	2891/2927 (98.8%)	1		1	
No	1488/1546 (96.2%)	3.13 (2.06–4.77)	<0.001	0.92 (0.50–1.68)	0.78
**Education (Years of schooling)**	9714/10037 (96.8%)	0.79 (0.75–0.83)	<0.001	0.83 (0.79–0.88)	<0.001
**Women’s age (years)**	9759/10084 (96.8%)	1.04 (1.02–1.05)	<0.001	1.02 (1.00–1.03)	0.02

ANC = Antenatal care; OR (95% CI) = Odds Ratio (95% Confidence Intervals)

Having experienced pregnancies in the past was significantly associated with greater odds of not attending antenatal care (crude OR = 2.44, 95% CI = 1.06–5.56) (Table 3). There was an age effect in this association though, as the age-adjusted OR was weaker and statistically non-significant (OR = 1.37, 95% CI = 0.56–3.45). Women who experienced past adverse pregnancy outcomes were also less likely to attend ANC, but this association was not statistically significant. The number of children was, however, significantly associated with an increase in the odds that women did not attend ANC (unadjusted OR = 1.27, 95% CI: 1.16–5.00) remaining significant even after adjusting for the women’s age (Table 3).

### Demographic and social determinants of place of delivery

Bivariate analysis showed that all the selected explanatory variables were associated with the place of delivery and most associations remained significant after adjusting for the presence of covariates (Table 5). Noteworthy is the increased risk of delivery outside a health facility in absence of ANC attendance (adjusted OR = 3.49, 95% CI = 2.57–4.74). Women’s odds of delivery outside a health facility was also higher for those living at greater distances and those living in rural areas (Table 5). Having more years of schooling, on the contrary, increased the likelihood of an institutionalised birth (Table 5).

**Table 5:** Adjusted and Unadjusted Odds Ratios (OR) of delivery at health facilities against predictor variables. Values of OR >1 indicate increased risk of delivery outside health facilities.

**Variables**	**n at health facility / n pregnancies (%)**	**Unadjusted OR (95% CI)**	***p***	**Adjusted OR (95% CI)**	***p***
**ANC attendance**					
Yes	4908/9733 (50.4%)	1		1	
No	52/321 (16.2%)	5.26 (3.9–7.1)	<0.001	3.49 (2.57–4.74)	<0.001
**Place of residence**					
Urban	4469/8491 (52.6%)	1		1	
Rural	493/1568 (31.4%)	2.42 (2.16–2.72)	<0.001	1.76 (1.51–2.04)	<0.001
**Distance to health facility**					
< 2 km	3841/7398 (52%)	1		1	
2–5 km	827/1607 (51%)	1.02 (0.91–1.14)	0.74	1.02 (0.92–1.14)	0.69
6–10 km	72/287 (25%)	3.23 (2.46–4.23)	<0.001	2.56 (1.94–3.37)	<0.001
11–15 km	68/220 (31%)	2.41 (1.81–3.22)	<0.001	1.11 (0.80–1.54)	0.52
>15 km	154/547 (28%)	2.76 (2.28–3.34)	<0.001	1.37 (1.08–1.73)	0.01
**Literacy**					
Yes	1750/2924 (59.8%)	1		1	
No	656/1538 (42.7%)	2.00 (1.77–2.27)	<0.001	3.32 (0.85–1.20)	0.93
**Education (Years of schooling)**	4941/10015 (49.3%)	0.9 (0.89–0.91)	<0.001	0.92 (0.90–0.93)	<0.001
**Women’s age (years)**	4962/10059 (49.3%)	1.01 (1.01–1.02)	<0.001	1.00 (1.00–1.01)	0.56

ANC = Antenatal care; OR (95% CI) = Odds Ratio (95% Confidence Intervals)

Past pregnancy experience was associated with greater odds that delivery takes place outside a health facility (crude OR = 2.94, 95% CI = 2.27–3.85), and this association remained significant (*p*<0.001) after adjusting for age (Table 3). Women who had experienced past adverse pregnancy outcomes were also less likely to deliver at a health facility, but this association was not significant (Table 3). The number of children was however significantly associated with the odds of a woman not delivering at a health facility (unadjusted OR = 1.18, 95% CI: 1.14–1.23), remaining significant after adjusting for the women’s age (Table 3).

## Discussion

We have used Dande HDSS 2009–2015 data, located in Bengo Province of Angola, about 60 km to the NE of Luanda, to identify factors that influence the utilization of health services by pregnant women and the associated risk factors for pregnancy outcome. There were 10,289 reported pregnancies, 98.5% of which resulted in live births, corresponding to about 15 deaths per 1,000 gestations. This compares with the perinatal mortality of 30 deaths per 1,000 gestations reported for Angola [11]. However, the perinatal mortality, includes live births with ensuing death within the first seven days of life [11], whereas the foetal deaths of our study focused exclusively on abortions and stillbirths. Nevertheless, we cannot exclude under-reporting of foetal deaths, as it has been previously documented for the Dande HDSS area [17] and in similar settings of low and middle-income countries [21–23]. Spontaneous and induced abortions are highly stigmatised. The first are related to social representations of women’s inability to have children, and the second are illegal. Therefore, abortions have probably been grossly under-reported in our study. Additionally, the lack of effective registration systems, cultural beliefs and stigma are the main obstacles to unbiased estimations of stillbirths and neonatal mortality [24]. Household surveys are very important sources of information in developing countries [23], but even this method does not keep people from omitting information because they don’t recognize the importance of reporting stillbirths or the death of infants who had not been previously registered [17, 20–22].

We have found that having at least one ANC visit, decreases the odds of a stillbirth and the odds of an abortion. These results corroborate the importance of ANC attendance for decreasing the risk of foetal death and are in line with a study conducted in Nigeria where, after controlling for variables similar to those we have studied, ANC attendance was the single significant predictor of live birth [25]. A study on ANC services and their implications for vital and health outcomes of children in 69 low and middle-income countries, also reported that the prevalence of newborn deaths was higher among women who did not attend ANC (3.12%) compared to those with at least one visit (1.67%) [26]. Several studies underline the benefits of ANC in the early detection of possible obstetric complications, treatment, and identification or modification of risk factors during pregnancy [2, 14, 27, 28]. Depending on the level of care, women attending ANC become more exposed to proper information, counselling and education about pregnancy, their health and child care. There is also a positive association between ANC attendance and later institutionalized delivery or use of SBA [29–31], again contributing to maternal and child mortality reduction [29, 32, 33, 34].

We have also found that in the Dande HDSS area, women who attended ANC were more likely to have an institutionalized delivery than otherwise. A similar association was found in Kenia [15, 35], Cambodia [36], Ghana [31, 37] and Bangladesh [38]. This prominence of the ANC appointment prompted us to examine the possible determinants of ANC attendance itself. The prevalence of ANC attendance was 96.8%, which is higher than the national figure of 81.6% [11], and higher than the reported median ANC of 89% in sub-Saharan Africa in 2010–2014 [29]. The number and timing of ANC visits of women participating in our study were in greater compliance with the WHO recommendations than those observed at the national level. In the Dande HDSS area, 82.5% of women had four or more visits and 63.7% had the first ANC contact within the first trimester of gestation, whereas in Angola those percentages were 61% and 40%, respectively [11]. In the Dande area, women may have attended ANC care to receive free medication, a mosquito bed net and a pregnancy card, which facilitates their access to hospital emergency in case of need, even if they were not planning an institutionalised delivery. Nevertheless, these perks probably do not explain the higher values of ANC prevalence when compared to those at national level. One possible explanation is the greater weight of urban women in our study (84.3%), given that in Angola the coverage of prenatal care is 92% in urban areas[11]. Also, 73.4% of households of our study were at less than 2 km from a health facility and this figure may be too optimist for Angola as a whole.

The use of ANC services is significantly influenced by the place where women live. In our study, the distance of the household to the nearest health facility was calculated using geographical information system methods. We have found that living far from a health facility is a major factor, as women living at distances greater than 2 km had an increased risk of missing ANC, compared to those at <2 km. Distance is a known barrier to health care utilization, as it is linked to lack of transport, poor access and costs [39–41]. In our study, women living in rural areas were also at greater risk of not attending any ANC than those in urban settings, and this remained true even after adjusting for distance to a health facility. Other studies also reported a decreased prevalence of ANC in rural areas [13, 42, 43]. The risk associated with distance accumulates with the risk of living in rural settings. The two factors combined, capture other aspects of remoteness such as poor road access, reduced communication between communities, disadvantaged socioeconomic status, adherence to cultural traditions, and limited access to information, among others [40].

Education has been commonly associated with an increased likelihood of health care utilization and is frequently pointed out as an important social determinant of maternal health care [15, 44, 45]. Educated women have more autonomy and capacity to make informed and responsible decisions about their health [45, 46]. They have a better understanding of the information conveyed by health professionals, for them and their children, namely regarding the importance of the continuum maternal care [45]. We have found that education is an important determinant of ANC attendance. One more year in school meant an average decrease in the odds of failing to attend ANC by OR = 0.83 (95% CI = 0.79–0.88), after adjusting for place of residence and age. This is in good agreement with the results of a recently published study conducted in Jordan [47], where the odds of non-utilization of ANC services decreases 0.87 (95% CI = 0.81–0.91) for an additional one-year in school [47]. This concurs with several other studies that found women’s education as a motivator and an important determinant of ANC attendance [15, 36, 39, 43, 48].

The influence of women’s age in maternal health care utilization is not linear and cannot be determined before an investigation, as different studies have found different lines of evidence [49]. The association between age and maternal health care utilisation might be related with different aspects: having been pregnant before, previous experiences in the use of ANC services, number of living children, cultural practices, health literacy, among others. In the Dande HDSS, older women had higher odds of not attending ANC visits [20, 50, 51], although other studies found the opposite [52–54]. Besides age, the women’s past obstetric experience was an important determinant of health services’ utilization in our study area. Generally speaking, older and more experienced women were less likely to use ANC services. Age by itself increased the odds of missing ANC by a factor of 1.02 per additional year of age, but the number of live children was also very influential, even after adjusting for the age effect. Having had a past adverse pregnancy outcome (stillbirth or abortion) did not change this picture, on the contrary, it appears to increase the odds of missing ANC, although this effect was not found significant, eventually due to lack of statistical power. There are a few possible explanations for these findings. On the one hand, older women, more likely to have past pregnancy experience, may have grown an increased feeling of being able to deal with another experience without institutional help. This feeling may become strong enough to make them decide to avoid the eventual inconveniences and costs of visits to the health facility. On the other hand, the quality of services provided during previous pregnancies is also likely to be influential. The acquired perception of how useful the services were in the past, can be crucial for the choices made in future pregnancies. We have not inquired on the women’s satisfaction regarding past health services utilization though, and further studies are needed to understand if these results are connected with previous pregnancies experiences.

Universal Health Coverage (UHC), under SDG 3, addresses different gaps in health care delivery [55]. An institutionalized delivery is more likely to provide safe conditions for both the mother and the newborn, thus contributing to prevent neonatal and maternal mortality [12, 29, 48], yet about 60% of births in Sub-Saharan Africa occur at home or in the absence of skilled birth attendants [12, 55]. In the Dande HDSS area, 50.7% of deliveries occurred outside a health facility. The likelihood of a non-institutionalised birth was greater for women who did not attend ANC and lived in rural areas. Corroborating previous studies [12, 48, 55], we have found that greater distance to a health facility was also associated with a non-institutionalized delivery. As for the women´s age and experience, the results were coincident with those mentioned above for the utilisation of ANC: more experienced women, with higher parity, were more likely to deliver outside health facilities, which is also in accordance with studies conducted elsewhere [13, 39, 48]. On the contrary, women with more years at school were more likely to give birth in a health facility, having a decreased odds of 0.92 per additional year in school [13, 30, 55].

The discrepancy between high levels of ANC attendance and low health facility delivery in the Dande HDSS area is in line with findings from studies conducted in other settings of Sub-Saharan Africa [32, 37, 56] and it may be related with the precipitous nature of women´s labour. Antenatal care (ANC) attendance does not demand a specific timing, but the imminence of delivery requires time for women to cover the distance to a health facility. The lack and cost of transportations, as well as poor road infrastructures, become thus involved in the decision on where deliver. In more experienced women (older, with previous pregnancies and with more children), self-confidence, and previous experiences might also be playing a key role in their final decision. A possible explanation is the added inconvenience of a temporary separation from the children, eventually brought about by a visit to the health facility. We have found that women already had an average of 3 live children when they were pregnant, with 39% having 4 or more children. The existence of support in the household for these children may be decisive. Future research should focus on the rationale underlying women´s choices and on the quality of care provided at health facilities, to understand what is constraining the use of health delivery services. Eventual poor quality of care and equipment, and women’s perception of that, is one of the hypotheses for abandoning the health system at the time of delivery.

The use of ANC services and health facilities proved to be positively associated with birth outcome. However, an important result from our study is the lack of equity in accessing these services. The main discriminant factors were the place of residence, namely the rural-urban dichotomy and distance to health facilities, and the women’s level of education. These determinants have been previously recognized as dimensions to equity in health services utilization [39] and are consistent with results reported by relevant studies in developing countries [13, 43, 44, 53]. We have also found that women’s age, which correlates with experience and number of children, in time, may also become an increasing obstacle to make use of health services. These later factors may result from a combination of factual difficulties with the women’s evolving evaluation of the benefits that they anticipate from using the services.

### Strength and limitations

This study was conducted within the scope of Dande HDSS activities, covering both urban and rural areas, involving the analysis of a six-year period (from 2009 to 2015), a large sample and a set of individual variables that allowed studying several sociodemographic determinants of maternal health care utilization. This study also focused on pregnancy outcomes that occurred in and outside health facilities, thus encompassing a better knowledge about maternal health care utilisation. To our knowledge, the few pieces of research conducted in Angola that approached determinants of maternal health care [52, 57] were hospital-based studies, therefore excluding women who did not use maternal health services and potential factors that kept them away from health facilities.

There is a wide range of factors that contribute to the utilisation of maternal health care. To assess them we have used a tool that continuously collects information on pregnancy results and allows access to indicators such as ANC attendance, number and timing of attendance, institutionalized births or access to SBA, and birth outcome. Despite the strengths of this study, it is important to acknowledge limitations, namely the probable under-reporting of events related to stillbirths, neonatal and perinatal deaths. Given the importance of data on foetal death to assess maternal and child care, it is extremely important to create reliable and permanent registration mechanisms that contribute to a better understanding of reality, especially in developing countries [3, 27]. The implementation of a pregnant surveillance system (PSS) and the follow-up of a birth cohort are currently being planned, enabling greater accuracy and a thorough knowledge of events concerning maternal and child health [17]. The PSS will enable the exploration of more than the individual determinants of access to maternal health care, namely the social context where women are embedded, and, for those attending ANC, the content, quality and the way interactions with health providers shape the continuum of maternal care. The 2016 WHO recommendations shifted the focus from coverage to content and recent research shows that the quality of ANC and delivery care, both important for the survival of mother and child, carry crucial importance in the use of maternal health care services [2, 3, 58].

An average of 6 months elapsed from pregnancy outcomes to women interviewing, which might still be considered appropriate to avoid recall bias. However, information bias particularly regarding adverse events cannot be completely ruled out. Our adverse events’ classification was based on women self-reports of gestational age (28 weeks being the discriminant timing) and these might not be accurate enough, even if women were answering at their best knowledge.

The economic and financial attributes of the household to which women belong might influence some of the variables studied. For example, access to education and transportation, are surely conditioned by economic status. Economic determinants are widely studied in the context of maternal care use in different settings. The construction of a wealth index with economic data of the households and the analyses of how it might influence relevant risk factors identified here is a logical next step currently under investigation, which we intend to publish in the future.

## Conclusions

Given that our study is based on data collected from 2009 to 2015, in this paper we refer to the WHO recommendation of a minimum of four ANC visits during pregnancy. In 2016, at the start of the SDGs, WHO launched new guidelines aiming ANC models with a minimum of eight contacts, with the first taking place between eight and twelve-weeks’ gestation. The current results suggest that there are difficulties in the implementation of more demanding models of access to maternal health care. Rich-poor and rural-urban gaps persist in women’s access to maternal health care services. Essential improvements are needed in the country’s capacity to address the determinants of maternal health and to adopt more appropriate interventions to local contexts. According to the results of our study, specific and articulated health and social policies are needed in Bengo Province, Angola, to address existing barriers to maternal health care, such as availability of proximity services, for instance increasing the number of health facilities, mainly in rural areas. Delivery facilities restricted mostly to urban areas may be preventing women from seeking institutionalized birth, given that place of residence and distance to health facilities are strongly associated with the place of delivery. Improvements in health assistance quality are also needed: in addition to trained and available human resources, maternal health care facilities should provide routine services endowed with basic essential obstetric care, not only capable of early detection and treatment of pregnancy problems but also of managing unexpected complications. Investment should be made in hospitals where there is at present lack of equipment and/or human resources, contributing for women to perceive health units as a safer and more propitious environment for childbirth, overcoming the barriers that have been preventing them from having an institutionalized birth. Referral systems and emergency transports, like ambulances, are essential in settings as Dande, where the lack of private or public transports is a constant.

These measures are critically dependent on the financial investment that authorities are willing to make in maternal health in Angola. However, to maximize their impact, and to reduce the gap between policies and reality on the ground, there is a need to strengthen leadership and governance capacity in the maternal health sector, and to develop information systems capable of informing and improving practices within the health system. Following a rigorous and decentralized reality-driven approach, systematic quality assessments of the services are required to help the identification of the main problems, to assure effective resource allocation, to make health services more socially accountable, and to develop solutions to improve maternal and child health services.

Social programmes aiming to reinforce women’s empowerment and education are also essential. Short-term measures, like awareness raising in communities and maternal education programmes are a priority, always involving different participants (women, families and health agents). Given the scarcity of health workers, and taking advantage of the existence of the civic organization Angolan Women Organization (OMA–Organização da Mulher Angolana) whose main objectives are to promote women's health care, legal education and the mediation of family conflicts, community-based initiatives aiming the strengthening of links between health services and women should be developed. OMA is already present in most of the neighbourhoods in the entire country and has a recognised social role both by authorities and by the community. Their involvement in the improvement of maternal health care could be based on a joint strategy with health services, assisting in health education, awareness raising among pregnant women, acting as mediators and facilitating a monitoring/tracking of women at antenatal care, delivery and postnatal care.

## Supporting information

S1 Text Questionnaire (English version). (DOCX)

S2 Text Questionnaire (Portuguese version). (DOCX)
