# Supplementary material for: Determinants of maternal health care and birth outcome in the Dande Health and Demographic Surveillance System area, Angola
Source: PLoS One. 2019 Aug 22;14(8):e0221280. doi: 10.1371/journal.pone.0221280 (PMC6706050; doi:10.1371/journal.pone.0221280)
Supplement: S1 Text — (DOCX) [file pone.0221280.s001.docx]

**S1 Text – Questionnaire (English version)**

**HDSS – Pregnancy outcome Form**

**Date of interview: Fieldworker:**

**Mother’s ID:** (Neighbourhood, Sector; House, Member)

**Woman’s name:**

**Pregnancy: Date of delivery:** Indicate if it is a date estimate or was confirmed with a document

**ANTENATAL CARE (ANC) ATTENDANCE**

**Did you have any ANC appointment during your last pregnancy?**

**Options:** Yes; No; Doesn't know/ Doesn't answer

**If YES: Where did you have ANC appointment?**

**Options:** Public; Private; If other - where?; Doesn't know/ Doesn't answer

**How many ANC appointments did you attend?**

**Options:** If mother doesn’t know the exact number, please fill the board.

| 1 |  |
| --- | --- |
| 2 or 3 |  |
| From 4 to 6 |  |
| From 7 to 9 |  |
| 10 or more |  |
| Doesn't know/ Doesn't answer |  |

**Roughly how many weeks/months pregnant were you when you had your first ANC appointment?**

**Options:** ____ weeks; ____ months; Doesn't know/ Doesn't answer

**PREGNANCY OUTCOME**

**Options:** Live birth; Stillbirth (late foetal death with gestation period of 28 or more weeks); Abortion (foetal death with less than 28 weeks of gestation)

**If live birth, indicate how many babies were born**

**Options:** One, twins, triplets, or more.

**Delivery place**

**Options:** Health centre; Maternal Health Centre or Hospital; Anywhere outside health facility

**If the delivery occurred Anywhere outside health facility, did you have any type of support?**

**Options:** Traditional midwife; Other support (family or relatives); No support at all

**Is this your first pregnancy?**

**Options:** Yes; No; Doesn't know/ Doesn't answer

**If NO, how many times have you been pregnant before?**

**Of those pregnancies, how many outcomes were live births (how many babies were born alive)?**
