# Supplementary material for: Determinants of maternal health care and birth outcome in the Dande Health and Demographic Surveillance System area, Angola
Source: PLoS One. 2019 Aug 22;14(8):e0221280. doi: 10.1371/journal.pone.0221280 (PMC6706050; doi:10.1371/journal.pone.0221280)
Supplement: S2 Text — (DOCX) [file pone.0221280.s002.docx]

**S2 Text – Questionnaire (Portuguese version)**
